# EFFECTIVENESS OF USING TECHNOLOGIES FOR COMMUNITY DWELLING OLDER ADULTS: A PILOT STUDY

**DOI:** 10.1093/geroni/igae098.2896

**Published:** 2024-12-31

**Authors:** Pei-Fen Chang, Lisa Thomas, Jeff Feng, Kai-Li Tsai

**Affiliations:** Texas Woman’s University Houston Campus, Houston, Texas, United States; The University of Texas Health Houston, Houston, Texas, United States; University of Houston, Houston, Texas, United States; Texas Woman’s University, Houston, Texas, United States

## Abstract

Many challenges arise with an aging population, particularly the increase of isolation and lack of social relationships. A strong sense of social connections is considered as a protective factor that is associated with general well-being. One novel approach that has been shown to increase social connection in older adults is the utilization of technology. During this post-pandemic period, we lack an understanding of whether or not older adults use technologies for daily living, especially for social connections. The aim of the study was to examine the effectiveness of an information and communication technology (ICT) program for community dwelling older adults for social connection by decreasing loneliness and increasing quality of life. A one-group pre and post-tests design was used including the inclusion criteria: older adults aged 70 and older, living alone in the community, and able to speak and understand English. Outcome measures included the SF-12 for physical and mental health, UCLA 3-item loneliness Scale for loneliness, and Technology Acceptance Model questionnaire for the self-perceived acceptance and ease of use of the technology. After receiving a 6-week intervention by using iPads, all participants demonstrated significant increase in quality of life and decreased loneliness scores. However, results of the self-perceived acceptance and ease of use of the technology were mixed. Results from this pilot study demonstrated that community dwelling older adults who live alone could benefit from using technology for communication to decrease loneliness and increase quality of life. Future study with larger sample size is needed.

